# SMonitoring the operational impact of insecticide usage for malaria control on *Anopheles funestus *from Mozambique

**DOI:** 10.1186/1475-2875-6-142

**Published:** 2007-10-31

**Authors:** Sonia LR Casimiro, Janet Hemingway, Brian L Sharp, Michael Coleman

**Affiliations:** 1 National Institute of Health, Av. Eduardo Mondlane/Salvador Allende, Maputo, Mozambique, P.O. Box 264; 2 Liverpool School of Tropical Medicine, Pembroke Place, Liverpool, UK, L3 5QA; 3 Malaria Research Programme, Medical Research Council, Ridge Road, Durban, South Africa

## Abstract

**Background:**

Indoor residual spraying (IRS) has again become popular for malaria control in Africa. This combined with the affirmation by WHO that DDT is appropriate for use in the absence of longer lasting insecticide formulations in some malaria endemic settings, has resulted in an increase in IRS with DDT as a major malaria vector control intervention in Africa. DDT was re-introduced into Mozambique's IRS programme in 2005 and is increasingly becoming the main insecticide used for malaria vector control in Mozambique. The selection of DDT as the insecticide of choice in Mozambique is evidence-based, taking account of the susceptibility of *Anopheles funestus *to all available insecticide choices, as well as operational costs of spraying.

Previously lambda cyhalothrin had replaced DDT in Mozambique in 1993. However, resistance appeared quickly to this insecticide and, in 2000, the pyrethroid was phased out and the carbamate bendiocarb introduced. Low level resistance was detected by biochemical assay to bendiocarb in 1999 in both *An. funestus *and *Anopheles arabiensis*, although this was not evident in WHO bioassays of the same population.

**Methods:**

Sentinel sites were established and monitored for insecticide resistance using WHO bioassays. These assays were conducted on 1–3 day old F1 offspring of field collected adult caught *An. funestus *females to determine levels of insecticide resistance in the malaria vector population. WHO biochemical assays were carried out to determine the frequency of insecticide resistance genes within the same population.

**Results:**

In surveys conducted between 2002 and 2006, low levels of bendiocarb resistance were detected in *An. funestus*, populations using WHO bioassays. This is probably due to significantly elevated levels of Acetylcholinesterase levels found in the same populations. Pyrethroid resistance was also detected in populations and linked to elevated levels of p450 monooxygenase activity. One site had shown reduction in pyrethroid resistance since the base line in 1999.

## Background

Malaria is a major cause of morbidity and mortality in Africa with an estimated 360 million clinical attacks [1] and 1–2 million deaths annually [2]. Malaria vector control relies on the use of effective insecticides, most commonly through indoor residual spraying (IRS) or insecticide-treated nets (ITN). The increased number of reports of insecticide resistant Anopheles species in Africa [3] is a threat to the success of insecticide-based malaria control programmes.

DDT was introduced for malaria control in 1943 [4] and was widely acclaimed due to its impact on reducing morbidity and mortality in malaria naïve troops in endemic regions in World War 2. Due to its success, DDT was rapidly introduced into public health and malaria control campaigns, and was the main insecticide used in the WHO malaria eradication campaign carried out between 1955 to 1969 [5]. Insecticide resistance in the vector is one of the major reasons given for the failure of the WHO campaign [5], but there is little evidence to support this claim in Africa [3,6].

The use of DDT was reduced from the 1970's with the introduction of pyrethroids. Its use in agriculture ceased internationally in the 1980s and there have been various attempts to ban its use completely since then. The Stockholm Convention on Persistent Organic Pollutants [29] seeks to ban persistent pollutants, including outdoor use of DDT in agriculture. However, due to the beneficial effects of DDT for malaria control the treaty contains an amendment which specifically authorises indoor use of DDT for vector control, subject to certain safeguards.

Pyrethroids, although an excellent insecticide class for controlling malaria, are only available in formulations with an accredited residual life of up to four months, requiring 2–3 rounds of IRS per year in endemic regions, compared to the 1–2 rounds of spray with DDT [7]. Pyrethroids remain the only class of insecticide available for the use on ITNs. As pyrethroid resistance has been selected, several control programmes, including Angola, South Africa, Mozambique and Zambia have reverted back to using DDT.

Early use of DDT also left the potential legacy of cross-resistance between DDT and pyrethroids through alterations in their common target site, the sodium channel [8], known as *kdr *resistance [9]. Most reports of *kdr *resistance in *Anopheles gambiae *come from West Africa where the use of DDT in agriculture probably contributed to the original selection and extensive spread of this resistance mechanism [10]. The only confirmed report in African Anopheles of *kdr *outside West Africa comes from Kenya, where a different mutation occurs changing the same amino acid residue in the sodium channel [11].

Whether *kdr *has an operational impact on ITNs has been tested in experimental field trials with conflicting results. An experimental hut trial in Côte d'Ivoire demonstrated a survival advantage for *kdr *resistant mosquitoes [12] and village randomized trials showed that ITNs continued to prevent malaria despite *kdr *resistance in the vector population. A recent study in Benin suggests that *kdr *is capable of undermining ITNs [13]. There is a real need to scale these studies up into malaria control programmes. The impact of *kdr *on IRS was significant in the malaria control programme on Bioko Island, Equatorial Guinea, as monitored through relative vector density resulting in a change from pyrethroid to carbamate for IRS [14]. Monitoring malaria cases in Kwa-Zulu Natal, South Africa, picked up the failure of pyrethroids in the IRS programme in the 1990s resluslting in DDT being reintroduced [15]. There are no reports of insecticide resistance not affecting an IRS programme.

Understanding the intricacies of resistance mechanisms, cross-resistance and impact on vectors is a pre-requisite for those involved in the selection of insecticides for and maintenance of large scale vector control programmes.

The Lubombo Spatial Development Initiative (LSDI) includes the southern most provinces of Mozambique. The LSDI, with sustained vector control and the use of effective drug treatment for malaria, has reduced *Plasmodium falciparum *prevalence rates from 88-65% to 33-4% at the 16 sentinel sites in Maputo province where entomological monitoring was completed [16]. The impact of the LSDI IRS programme reduced the numbers of vector species over time making mosquito collections increasingly difficult. It has been established that the impact of a successful IRS programme may eradicate *Anopheles funestus *from an area [15,17]. In central Mozambique, outside the LSDI area, control has primarily been through the distribution of pyrethroid impregnated bednets, although IRS with DDT is now being introduced in central provinces as part of the National Malaria Control Programme (NMCP).

The objective of this work is to determine if monitoring insecticide resistance is feasible for a malaria control programme and what the impact of monitoring will have on policy.

## Methods

### Field collections

**Figure 1:**
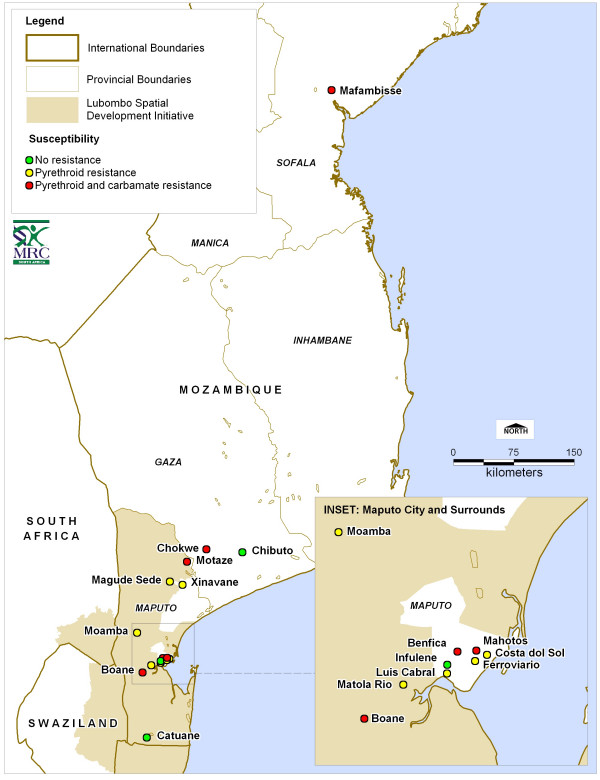
Insecticide resistance status at twenty localities for *An*. funestus in Mozambique.

Indoor resting blood-fed adult female *An. funestus *were collected in houses, using an aspirator, during the hours of 06.00–10.00 between Aug 2002 and June 2006, from sixteen localities in Mozambique (Figure 1). These collections occurred during the last four years of the Lubombo Spatial Development Initiative malaria control programme in southern Mozambique. The female mosquitoes were transported to the laboratory of the National Institute of Health in Maputo, kept in individual oviposition tubes and allowed to lay eggs. Families were reared separately through to 1–3 day old F1 adults at 26°C +/- 2°C and 70–80% RH. Wild caught mosquitoes were not analysed directly as exposure to insecticides that may have occurred prior to their capture might bias results. The use of 1–3 day olds from the sugar-fed F1 adults for all experiments allowed standardisation of age, physiological state and testing conditions for all assays.

### Species identification

Wild-caught females were morphologically identified as belonging to the *An. funestus *complex [18,19] and sibling species were identified using the ribosomal DNA-polymerase chain reaction [20].

### Insecticide susceptibility assays

Insecticide susceptibility assays were carried out following the WHO protocol [21] on a random sample of adult mosquitoes from each family. Between five and 25 adult F1 male and female mosquitoes were exposed to insecticide treated or control papers for 1 hour and then held in holding tubes with access to 10% sugar solution for 24 hours before the percentage mortality was determined. The insecticides tested were lambda-cyhalothrin (0.05%), deltramethrin (0.05%), bendiocarb (0.01%), and DDT (4%). All insecticide papers were supplied by WHO. Chi square and Fishers Exact tests were used to compare insecticide susceptibility assay results over time from the same locality.

### Biochemical assays

Biochemical assays were carried out on individual mosquitoes from the same family. Altered acetylcholinesterase (AChE) susceptibility to inhibition, activity levels of glutathione-*S *transferase (GST) and general esterases (α- and β-naphthyl acetate) and quantities of monooxygenase (p450) were estimated and values corrected for protein concentration as described by Penilla *et al*. (1998) [22]. The insecticide susceptible Durban (DurbanS) laboratory strain of *Anopheles arabiensis *was used as a reference strain. This strain has been maintained in the laboratory for 12 years without exposure to insecticides.

Two-sample t-tests were used to compare the results of the biochemical assays between the standard susceptible DurbanS strain and the field samples and to look for correlation between the biochemical assays and bioassay results.

## Results

The location of sixteen collection sites in Mozambique from which *An*. funestus were collected between 2002–2006 are shown in Figure 1. A total of 4,162 one to three-day old F1 adult progeny were reared from the wild-caught adult *An. funestus *females collected, from these localities for subsequent bioassays and biochemical assays.

Six of the sixteen sites (Benfica, Boane, Catuane, Chokwe, Mafambisse and Moamba) were included in the original baseline study in 1999 [23]. In 2006, as mosquito numbers were limited from each family, priority was given to testing lambda-cyhalothrin and bendiocarb, as these were the insecticides in use in the malaria control programme. All sites were tested with lambda-cyhalothrin, eleven with bendiocarb, three with DDT and four with deltamethrin (Table 1). Low level resistance to bendiocarb was detected at Benfica, Boane, Chokwe, Mafambisse, Mahotas and Motaze. These sites also had resistance to lambda-cyhalothrin and deltamethrin. No resistance was detected to DDT.

**Table 1:** WHO susceptibility test results on 1-3 day old F1 *An. funestus * from  16 localities in Mozambique 2006 data with Chi square comparisons  to  6 of the study sites from the original 1999 base line survey. (- No data available)

Locality	Latest data 2002 to 2006								Base line data from 1999					
	
	Lambda-Cyhalothrin (0.05%)		Delta-methrin (0.05%)		Bendiocarb (0.01%)		DDT (4%)		Lambda-Cyhalothrin (0.05%)		Delta-methrin (0.05%)		Bendiocarb (0.01%)	
	
	n	M	n	M	n	M	n	M	n	M	n	M	n	M
Benfica	240	94	138	90	220	99	-	-	19	100_a_	16	43.8_b_	16	100_a_
Boane	426	92	25	96	372	98	-	-	741	46.2_b_	302	98.2_a_	449	97.3_a_
Catuane	34	100	-	-	-	-	-	-	44	72.7_b_	-	-	-	-
Chibuto	48	100	-	-	59	100	-	-	-	-	-	-	-	-
Chokwe	131	84	-	-	108	96	-	-	12	100_a_	-	-	16	100_a_
Costa dol Sol	70	81	-	-	-	-	-	-	-	-	-	-	-	-
Ferroviario	21	76	-	-	-	-	-	-	-	-	-	-	-	-
Infulene	14	100	-	-	38	100	-	-	-	-	-	-	-	-
Luis Cabral	20	90	-	-	-	-	-	-	-	-	-	-	-	-
Mafambisse	139	95	-	-	149	95	68	100	23	100_a_	11	-	22	100_a_
Magude Sede	238	88	-	-	150	100	23	100	-	-	-	-	-	-
Mahotas	55	96	17	88	33	99	-	-	-	-	-	-	-	-
Matola	261	90	-	-	209	100	-	-	-	-	-	-	-	-
Moamba	29	83	25	96	-	-	-	-	87	75_a_	109	83.5_a_	-	-
Motaze	435	83	-	-	300	97	14	100	-	-	-	-	-	-
Xinavane	23	83	-	-	12	100	-	-	-	-	-	-	-	-

M = percentage mortality _a _= p > 0.1 _b _= p < 0.001

Significant increase in pyrethroid resistance was detected in Benfica, Boane, Catuane, Chokwe and Moamba (p < 0.05). Other sites, e.g. Mahotas, also showed increases in pyrethroid resistance, although the significance of the rise is unknown as the sample sizes (n<30) were low. A significant decrease (P < 0.001) in pyrethroid resistance was recorded at Catuane, where baseline mortality was 72.7% which increased to 100% susceptibility in 2006.

Mosquitoes from all sites tested had significantly higher p450 levels compared to the Durban susceptible strain (Table 2). Increased p450 activity has already been suggested as the pyrethroid resistance mechanism in *An. funestus *from Mozambique [23,24].

**Table 2:** Comparisons of average values for a range of biochemical assays between F1 adult progeny *An. funestus *from field populations and the *An. arabiensis *Durban insecticide susceptible reference strain.

Area	α-naphthyl acetate		β-naphthyl acetate		p450		GST		AChE		No. tested
	
	ave.	**s.d**	ave.	**s.d**	ave.	**s.d**	ave.	**s.d**	% inhib.	**s.d**	
Benfica	0.083	0.045	0.36	0.045	1.65	0.11	0.08	0.02	62_a_	10.3	135
Boane	0.064	0.04	0.27	0.135	1.68	1.11	0.07	0.03	-	-	44
Catuane	0.058	0.037	0.3	0.131	2.26	1.34	0.07	0.02	-	-	89
Cotsuane	0.028	0.012	0.101	0.047	1.15	0.067	0.06	0.02	62_a_	12.6	121
Ferroviaro	0.09	0.029	0.397	0.104	1.15	0.456	0.06	0.017	72_a_	10.2	29
Infulene	0.108	0.065	0.593	0.286	1.4	0.39	0.07	0.019	72_a_	9.1	31
Magude Sede	0.103	0.072	0.414	0.241	1.7	0.68	0.05	0.012	75_a_	10	40
Manguiza	0.064	0.045	0.277	0.125	1.86	0.838	0.06	0.2	67_a_	15.6	159
Matola	0.08	0.059	0.36	0.23	1.4	0.55	0.067	0.026	71_a_	11.1	208
Motaze	0.081	0.05	0.321	0.18	2.3	1.11	0.069	0.018	71_a_	13.3	27
DurbanS	1.0	0.17	0.64	0.23	.56	0.25	0.14	0.11	98_a_	4.25	100

α- naphthyl acetate: mMoles α-naphthol produced/min/mg protein. β- naphthyl acetate: mMoles β-naphthol produced/min/mg protein. p450: Equivalent units of cytochrome p450. GST: GST activity. AChE: average percentage inhibition of AChE by propoxur. a: those populations showing a significant difference, P < 0.001, to the DurbanS susceptible laboratory strain that may confer insecticide resistance.

The original baseline survey showed low levels of carbamate resistance that were associated with low levels of an altered AChE [23]. A high level of altered AChE resistance frequency was observed at all sites tested (Table 2). This mechanism is the probable cause of the low levels of carbamate resistance observed in bioassays.

No increased levels of esterase or GST activity were detected in *An. funestus *from any locality tested compared to the Durban susceptible strain.

## Discussion

The development of insecticide resistance is a potential threat to any insecticide-based malaria vector control programme. The number of insecticides and formulations recommended by the WHO Pesticide Evaluation Scheme (WHOPES) for IRS is severely limited [25]. This arsenal may be further depleted by the lack of local country or regional insecticide registrations. To ensure that the insecticides used for IRS in Mozambique remain effective and their choice is evidence-based, an assessment of the resistance profile and potential resistance mechanisms within the targeted vector populations needs to be routinely monitored. Since the original baseline established in 1999 [23], the resistance profile has been monitored in sixteen localities using WHO bioassays and in ten of these localities using biochemical assays to assess potential resistance mechanisms.

Previously the NMCP in Mozambique used DDT before a change in policy in 1993 when the pyrethroid lambda-cyhalothrin was introduced. In 1999 when the baseline survey was undertaken, both *An. funestus *and *An*. *arabiensis *were resistant to lambda- cyhalothrin [23,26]. The same resistance profile was detected in *An. funestus *in Kwa-Zulu Natal, South Africa, which borders southern Mozambique. The onset of measurable insecticide resistance selection correlated with a surge in malaria in that region [27]. Pyrethroid resistance in *An. funestus*, in this region was correlated with increased titres of p450 [23,24]. The detection of resistance prompted a change of insecticide in the LSDI programme, the carbamate bendiocarb replacing lambda-cyhalothrin during the 2000 spray season [16]. Bendiocarb was then sprayed bi-annually until 2005, while resistance to the three insecticide classes registered in Mozambique (carbamates, pyrethroids and latterly DDT) were monitored in an attempt to establish a resistance management plan to ensure sustainability of the programme.

Low levels of bendiocarb resistance were detected in *An. funestus *in the original 1999 baseline [23]. Resistance was still detectable by bioassay and associated with high frequencies of an altered AChE resistance mechanism in the 2002–06 collections, which is the likely cause of this resistance. This, coupled with the appearance of carbamate resistance in Mozambican *An. arabiensis*, the second malaria vector in the region (Coleman et al in press), and the high economic costs associated with bendiocarb use, prompted an operational change of insecticide in 2006 back to DDT. The levels of pyrethroid resistance still segregating in *An. funestus *at this point were considered too high to justify a switch back to pyrethroid treatment.

The decline in pyrethroid resistance at some sites suggests that with the correct resistance management strategy in place, pyrethroids may again play a role in southern Mozambique's malaria control programmes [28].

## Conclusion

This work demonstrates that operationally incorporating monitoring of insecticide resistance in space and time is feasible. The ability to use this data to develop evidence-based insecticide resistance management strategies that tie in with good monitoring of the impact of vector control operations on disease is also essential for sustainable large scale insecticide-based malaria vector control. The monitoring effort reported here resulted in National Malaria Control Campaign policy changes from pyrethroid to carbamate [23,26] to DDT over time. This informed decision making will ultimately have cost savings for the malaria control programmes in Mozambique. Continual monitoring will also allow for the establishment of insecticide resistance management programmes, protecting the limited number of insecticides available for malaria control.

## Authors' contributions

SC carried out all the field work and laboratory work. MC completed the analysis and draft manuscript. JH corrected the manuscript and guided analysis. BS conceived the initial ideas with JH.

## References

[B1] Snow RW, Guerra CA, Noor AM, Myint HY, Hay SI (2005). The global distribution of clinical episodes of Plasmodium falciparum malaria. Nature.

[B2] Breman JG, Alilio MS, Mills A (2004). Conquering the intolerable burden of malaria: what's new, what's needed: a summary. Am J Trop Med Hyg.

[B3] Coleman M, Sharp B, Seocharan I, Hemingway J (2006). Developing an evidence-based decision support system for rational insecticide choice in the control of African malaria vectors. J Med Entomol.

[B4] Gahan JB, Travis BV, Morton PA, Lindquist AW (1945). DDT as a Residual-Type Treatment to Control Anopheles quadrimaculatus. Econ Entom.

[B5] Trigg PI, Kondrachine AV (1998). Commentary: malaria control in the 1990s. Bull World Health Organ.

[B6] Coetzee M, Horne D, Brooke BD, Hunt RH (1999). DDT, dieldrin and pyrethroid insecticide resistance in African malaria vector mosquitoes: an historical review and implications for future malaria control in Southern Africa.. South African Journal of Science.

[B7] Najera JA, Zaim M (2002). Malaria Vector Control. Decision Making Criteria and Procedures for Judicious use of Insecticides.

[B8] Soderlund DM, Bloomquist JR (1989). Neurotoxic actions of pyrethroid insecticides. Annu Rev Entomol.

[B9] Martinez-Torres D, Chandre F, Williamson MS, Darriet F, Berge JB, Devonshire AL, Guillet P, Pasteur N, Pauron D (1998). Molecular characterization of pyrethroid knockdown resistance (kdr) in the major malaria vector Anopheles gambiae s.s. Insect Mol Biol.

[B10] Mouchet J (1988). Mini review:agriculture and vector resistance.. Insect Sci App.

[B11] Ranson H, Jensen B, Vulule JM, Wang X, Hemingway J, Collins FH (2000). Identification of a point mutation in the voltage-gated sodium channel gene of Kenyan Anopheles gambiae associated with resistance to DDT and pyrethroids. Insect Mol Biol.

[B12] Kolaczinski JH, Fanello C, Herve JP, Conway DJ, Carnevale P, Curtis CF (2000). Experimental and molecular genetic analysis of the impact of pyrethroid and non-pyrethroid insecticide impregnated bednets for mosquito control in an area of pyrethroid resistance. Bull Entomol Res.

[B13] N'Guessan R, Corbel V, Akogbeto M, Rowland M (2007). Reduced efficacy of insecticide-treated nets and indoor residual spraying for malaria control in pyrethroid resistance area, Benin. Emerg Infect Dis.

[B14] Sharp BL, Ridl FC, Govender D, Kuklinski J, Kleinschmidt I (2007). Malaria vector control by indoor residual insecticide spraying on the tropical island of Bioko, Equatorial Guinea. Malar J.

[B15] Maharaj R, Mthembu DJ, Sharp BL (2005). Impact of DDT re-introduction on malaria transmission in KwaZulu-Natal. S Afr Med J.

[B16] Sharp BL, Kleinschmidt I, Streat E, Maharaj R, Barnes KI, Durrheim DN, Ridl FC, Morris N, Seocharan I, Kunene S, La Grange JJ, Mthembu JD, Maartens F, Martin CL, Barreto A (2007). Seven years of regional malaria control collaboration--Mozambique, South Africa, and Swaziland. Am J Trop Med Hyg.

[B17] B. M (1986). The control of malaria with special reference to the contributions made by the staff of the South African Institute for Medical Research. S Afr Med J.

[B18] Gillies MT, DeMeillon B (1968). The Anophelinae of Africa South of the Sahara.

[B19] Gillies MT, Coetzee M (1987). A supplement to: The Anophelinae of Africa South of the Sahara. The Anophelinae of Africa South of the Sahara.

[B20] Koekemoer LL, Kamau L, Hunt RH, Coetzee M (2002). A cocktail polymerase chain reaction assay to identify members of the Anopheles funestus (Diptera: Culicidae) group. Am J Trop Med Hyg.

[B21] WHO (1998). Test Procedures for Insecticide Resistance Monitoring in Malaria Vectors, Bio-Efficacy and Persistence of Insecticides on Treated Surfaces.. Report of the WHO informal Consultation, 28-30 September.

[B22] Penilla RP, Rodriguez AD, Hemingway J, Torres JL, Arredondo-Jimenez JI, Rodriguez MH (1998). Resistance management strategies in malaria vector mosquito control. Baseline data for a large-scale field trial against Anopheles albimanus in Mexico. Med Vet Entomol.

[B23] Casimiro S, Coleman M, Mohloai P, Hemingway J, Sharp B (2006). Insecticide resistance in Anopheles funestus (Diptera: Culicidae) from Mozambique. J Med Entomol.

[B24] Brooke BD, Kloke G, Hunt RH, Koekemoer LL, Temu EA, Taylor ME, Small G, Hemingway J, Coetzee M (2001). Bioassay and biochemical analyses of insecticide resistance in southern African Anopheles funestus (Diptera: Culicidae).. Bull Entomol Res.

[B25] W.H.O (2001). Chemistry and specifications of pesticides. World Health Organ Tech Rep Ser.

[B26] Casimiro S, Coleman M, Hemingway J, Sharp B (2006). Insecticide resistance in Anopheles arabiensis and Anopheles gambiae from Mozambique. J Med Entomol.

[B27] Hargreaves K, Koekemoer LL, Brooke BD, Hunt RH, Mthembu J, Coetzee M (2000). Anopheles funestus resistant to pyrethroid insecticides in South Africa. Med Vet Entomol.

[B28] Hemingway J, Penilla RP, Rodriguez AD, James BM, Edge W, Rogers H, Rodrigez M (1997). Resistance management strategies in malaria vector mosquito control. A large-scale field trial in Southern Mexico.. Pestic Sci.

[B29] http://www.pops.int.

