# Monitoring the operational impact of insecticide usage for malaria control on *Anopheles funestus *from Mozambique

**DOI:** 10.1186/1475-2875-7-8

**Published:** 2008-01-09

**Authors:** Sonia LR Casimiro, Janet Hemingway, Brian L Sharp, Michael Coleman

**Affiliations:** 1 National Institute of Health, Av. Eduardo Mondlane/Salvador Allende, PO Box 264, Maputo, Mozambique; 2 Liverpool School of Tropical Medicine, Pembroke Place, Liverpool, L3 5QA, UK; 3 Malaria Research Programme, Medical Research Council, Ridge Road, Durban, South Africa

Since publication of our article [1], we have been made aware of several errors in our article.

In the Background section, paragraph 4, the first sentence "Pyrethroids, although an excellent insecticide class for controlling malaria, are only available in formulations with an accredited residual life of up to four months" should read "Pyrethroids, although an excellent insecticide class for controlling malaria, are only available in formulations with an accredited residual life of between 3 to 6 months [2]."

In the Background section, paragraph 4, the last sentence "As pyrethroid resistance has been selected, several control programmes, including Angola, South Africa, Mozambique and Zambia have reverted back to using DDT." should read "As pyrethroid resistance has been selected, several control programmes, including South Africa and Mozambique have reverted back to using DDT. Where cross-resistance occurs as in Equatorial Guinea alternative insecticide groups are being used."

In the Background section, paragraph 5, the last sentence "The only confirmed report in African Anopheles of kdr outside West Africa comes from Kenya, where a different mutation occurs changing the same amino acid residue in the sodium channel [11]." should read "Until recently, the only confirmed report in African Anopheles of kdr outside West Africa came from Kenya, where a different mutation occurs changing the same amino acid residue in the sodium channel [11]."

In the Results section, paragraph 2, the first sentence "Five of the twenty sites (Benfica, Boane..." should read"Six of the sixteen sites (Benfica, Boane...".

In the Results section, paragraph 3, "Significant increase in pyrethroid resistance was detected in Benfica, Boane, Catuane, Chokwe and Moamba (p < 0.05). Other sites, e.g. Mahotas, also showed increases in pyrethroid resistance, although the significance of the rise is unknown as the sample sizes (n < 30) were low. A significant decrease (P < 0.001) in pyrethroid resistance was recorded at Catuane, where baseline mortality was 72.7% which increased to 100% susceptibility in 2006." should read "Significant decreases in pyrethroid resistance were detected in Benfica, Boane, Catuane, Chokwe and Moamba (p < 0.05). Other sites, e.g. Mafambisse, showed increases in pyrethroid resistance, although the significance of the rise is unknown as the sample sizes (n < 30) were low."

In addition the legend of Table 1 should read.

**Table 1:** WHO susceptibility test results on 1–3 day old F1 An. Funestus from 16 localities in Mozambique 2006 data with Chi square comparisons to 6 of the study sites from the original 1999 base line survey. (- No data available)

Locality	Latest data 2002 to 2006								Base line data from 1999					
	
	Lambda-Cyhalothrin (0.05%)		Delta-methrin (0.05%)		Bendiocarb (0.01%)		DDT (4%)		Lambda-Cyhalothrin (0.05%)		Delta-methrin (0.05%)		Bendiocarb (0.01%)	
	
	n	M	n	M	n	M	n	M	n	M	n	M	n	M
Benfica	240	94	138	90	220	99	-	-	19	100_a_	16	43.8_b_	16	100_a_
Boane	426	92	25	96	372	98	-	-	741	46.2_b_	302	98.2_a_	449	97.3_a_
Catuane	34	100	-	-	-	-	-	-	44	72.7_b_	-	-	-	-
Chibuto	48	100	-	-	59	100	-	-	-	-	-	-	-	-
Chokwe	131	84	-	-	108	96	-	-	12	100_a_	-	-	16	100_a_
Costa dol Sol	70	81	-	-	-	-	-	-	-	-	-	-	-	-
Ferroviario	21	76	-	-	-	-	-	-	-	-	-	-	-	-
Infulene	14	100	-	-	38	100	-	-	-	-	-	-	-	-
Luis Cabral	20	90	-	-	-	-	-	-	-	-	-	-	-	-
Mafambisse	139	95	-	-	149	95	68	100	23	100_a_	11	-	22	100_a_
Magude Sede	238	88	-	-	150	100	23	100	-	-	-	-	-	-
Mahotas	55	96	17	88	33	99	-	-	-	-	-	-	-	-
Matola	261	90	-	-	209	100	-	-	-	-	-	-	-	-
Moamba	29	83	25	96	-	-	-	-	87	75_a_	109	83.5_a_	-	-
Motaze	435	83	-	-	300	97	14	100	-	-	-	-	-	-
Xinavane	23	83	-	-	12	100	-	-	-	-	-	-	-	-

M = percentage mortality _a _= p > 0.1 _b _= p < 0.001

Table 1. WHO susceptibility test results on 1–3 day old F1 *An. funestus *from 16 localities in Mozambique 2006 data with Chi square comparisons to six of the study sites from the original 1999 base line survey. (- No data available)

The corrected version of Table 1 is given here.

We apologize for any inconvenience or confusion that this may have caused.

## References

[B1] Casimiro S, Hemingway J, Sharp B, Coleman M (2007). Monitoring the operational impact of insecticide usage for malaria control on *Anopheles funestus *from Mozambique. Malaria J.

[B2] WHO Pesticides Evaluation Scheme. http://www.who.int/whopes/quality/en/.

